# Long Noncoding RNA in Myeloid and Lymphoid Cell Differentiation, Polarization and Function

**DOI:** 10.3390/cells9020269

**Published:** 2020-01-22

**Authors:** Imran Ahmad, Araceli Valverde, Fayek Ahmad, Afsar Raza Naqvi

**Affiliations:** Mucosal Immunology Lab, College of Dentistry, University of Illinois at Chicago, Chicago 60612, IL, USA; iahmad@uic.edu (I.A.); avalverd@uic.edu (A.V.); fahmad21@uic.edu (F.A.)

**Keywords:** long noncoding RNA, myeloid cells, lymphoid cells, immune responses, polarization

## Abstract

Long noncoding RNA (lncRNA) are a class of endogenous, non-protein coding RNAs that are increasingly being associated with various cellular functions and diseases. Yet, despite their ubiquity and abundance, only a minute fraction of these molecules has an assigned function. LncRNAs show tissue-, cell-, and developmental stage-specific expression, and are differentially expressed under physiological or pathological conditions. The role of lncRNAs in the lineage commitment of immune cells and shaping immune responses is becoming evident. Myeloid cells and lymphoid cells are two major classes of immune systems that work in concert to initiate and amplify innate and adaptive immunity in vertebrates. In this review, we provide mechanistic roles of lncRNA through which these noncoding RNAs can directly participate in the differentiation, polarization, and activation of myeloid (monocyte, macrophage, and dendritic cells) and lymphoid cells (T cells, B cells, and NK cells). While our knowledge on the role of lncRNA in immune cell differentiation and function has improved in the past decade, further studies are required to unravel the biological role of lncRNAs and identify novel mechanisms of lncRNA functions in immune cells. Harnessing the regulatory potential of lncRNAs can provide novel diagnostic and therapeutic targets in treating immune cell related diseases.

## 1. Introduction

Comprehensive analysis of human transcriptome revealed that our genome is pervasively transcribed [1]. However, compared to previous estimates of more than 100,000 protein-coding genes encoded by the human genome, genomic and functional analysis only validated 20,000 protein-coding genes [2]. This implies that only around 2% of the total human genome sequence is protein coding and the remaining genome transcribes noncoding RNA, govern transcriptional regulation or is transcriptionally inactive. Noncoding RNAs do not code for proteins and include transfer RNA (tRNA), small nuclear RNA (snRNA), small nucleolar RNA (snoRNA), ribosomal RNA (rRNA), and long non-coding RNA (lncRNA). Genes transcribing regulatory RNAs share almost equivalent mammalian genome landscape as that of protein coding genes. 

LncRNAs constitute most of this non-coding genomic space. These are classified as transcripts that are longer than 200 nucleotides in length and do not code for proteins. LncRNA genes have their own promoters, transcription factor binding sites and preferred transcription factors [3]. LncRNA are transcribed by RNA-polymerase II from different regions throughout the genome. The maturing lncRNA is then polyadenylated at the 3’-end and a methyl-guanosine cap is added to the 5’-end [4]. However, there are some lncRNAs such as BC200 and as-Oct4-pg5 which are not polyadenylated [5,6]. As of now, almost 17,000 lncRNA loci producing almost 50,000 isoforms have been identified in humans and the list continue to expand (Ensembl release v98). Based on their genomic location, lncRNAs can be classified as overlapping when intersecting a coding gene, divergent when the lncRNA is produced from a coding gene promoter region in reverse orientation, intronic when the entire lncRNA sequence is within the intron of a coding gene, intergenic when the lncRNA sequence is between two genes, or sense or antisense if the lncRNA is mapped between one or more exons of another transcript on the same (sense) or opposite (antisense) strand [3,4,7].

Unlike protein coding genes, lncRNAs exhibit high tissue specificity, poor sequence conservation, and relatively lower expression [7]. However, the act of transcription and limited conservation of short sequences is sufficient for lncRNA functionality across the species, but this prediction is difficult in distant species [7]. LncRNAs are distributed in the nuclear and/or cytoplasmic compartments, while mRNAs are predominantly present in cytoplasm indicating a broader regulatory role for lncRNAs. These regulatory RNAs work as modular molecules with individual domains. The capability of lncRNAs to physically interact with DNA, RNA or proteins allows them to regulate transcriptional, post-transcriptional, and translational mechanisms. Aberrations in lncRNA expression have been associated with disease manifestation in cancers, neurodegenerative disorders, autoimmunity, etc. [7,8,9,10].

By virtue of their multiple functions in recruiting transcription factors (TF), acting as microRNA sponges, translational regulation of various mRNAs, transcriptional silencing, and epigenetic reprogramming, lncRNAs participate in numerous biological pathways encompassing cell growth to apoptosis [11,12,13,14]. The massive number of lncRNAs expressed in different cell types signifies their extraordinary functional role in cellular processes. However, the studies in this field are still in their infancy with regard to their functions in immune cells. In terms of functional annotation, however, lncRNAs are beyond our understanding; an unknown, undiscovered RNA world that we encode but their functional decoding remains a mystery.

**Figure 1:**
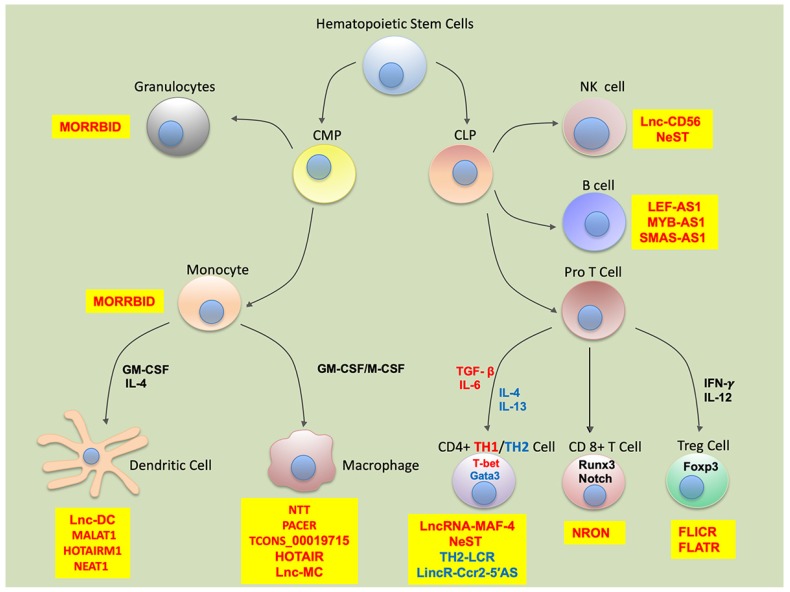
Schematic representation of the long noncoding RNA-mediated regulation of myeloid and lymphoid cell differentiation and function. Hematopoietic stem cells differentiate into various myeloid and lymphoid cells under the influence of molecular factors including cytokines and transcription factors. Several long noncoding RNAs have been characterized with a critical role in myeloid and lymphoid cell differentiation and function. LncRNAs with unique regulatory roles in myeloid or lymphoid cell-specific functions are listed in yellow boxes. In CD4+ differentiation, blue and red font corresponds to Th2 and Th1 cells, polarizing cytokines, or transcription factors. CMP: common myeloid progenitor; CLP: common lymphoid progenitor; Th: T helper; NK cell: Natural Killer Cell.

Hematopoietic Stem Cells (HSCs) are the stem cells from which all types of blood cells originate through a series of lineage committed differentiation steps which results in progressive loss of differentiation potential. White blood cells are broadly categorized as lymphoid or myeloid in lineage. Lymphoid cells include T cells, B cells and NK (Natural Killer) cells, while myeloid cells include monocytes, macrophages, dendritic cells, erythrocytes, basophils, neutrophils, eosinophils, and megakaryocytes [15]. LncRNAs have been found in various immune cells including macrophages, dendritic cells, neutrophils, basophils, eosinophils, and lymphoid cells. The development, differentiation, and activation of various immune cells has been found to be linked with the expression levels of certain lncRNAs. Numerous differentiation-associated lncRNAs have been identified and a major task is to uncover their functional roles. Although lncRNAs are generally considered to be low copy number transcripts, some of the lncRNAs can have transcript levels similar to highly expressed protein-coding genes or miRNAs. LncRNAs can rapidly shape transcriptional, post-transcriptional and translational outputs and facilitate cells to polarize promptly depending on the inflammatory conditions. In this review, we will provide an update on the lncRNAs that have been characterized in regulating myeloid and lymphoid cell differentiation, polarization, and the activation of immune response. We will highlight how lncRNAs work in concert with transcription factors and microRNAs to acquire immune cell phenotype. Figure 1 and Table 1 shows a list of lncRNAs specific to each immune cell type and effector cell function. 

## 2. Myeloid Cells

**Figure 2:**
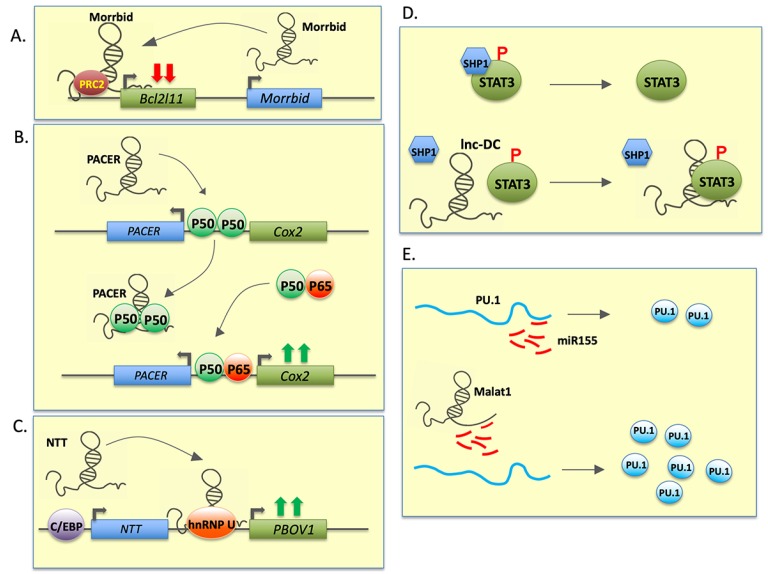
Schematic diagram depicting different long noncoding RNA mechanisms in myeloid cells. LncRNAs are modular molecule and can act as decoys, signals, guides, or scaffolds. In this figure, different characterized mechanisms through which lncRNAs exert their biological functions are listed. Examples of few lncRNA-mediated mechanisms that govern biological pathways in (**A**–**C**) macrophages or (**D**–**E**) dendritic cells are provided. (**A**–**C**) In macrophages, Morrbid, PACER, and NTT recruit specific transcription factors or ribonucleoproteins and regulate target gene expression. (**D**–**E**) Lnc-DC prevent SHP1-mediated dephosphorylation of dendritic cells specific transcription factor STAT3 to facilitate cell differentiation, while MALAT1 sequesters PU.1-targeting miR-155 and enhances cellular PU.1 levels. Green arrows denote transcriptional activation and red arrows denote transcriptional repression.

LncRNAs have been demonstrated to regulate differentiation and function of various immune cells. Recent studies have shown that some lncRNAs can integrate extracellular inputs with chromatin modification pathways, which ultimately allow cells to adapt to their environment rapidly [16]. Myeloid RNA regulator of Bim-induced death (Morrbid) is a conserved, nuclear localized lncRNA that was identified by Henoa-Mejia and colleagues as a highly and selectively expressed transcript in mature short-lived myeloid cells [17]. Morrbid-deficient mice display defects in short-lived myeloid cells (eosinophils, neutrophils and Ly6Chi monocytes), but no other myeloid or lymphoid cells. Evidently, these mice were susceptible to bacterial (*Listeria monocytogenes*) infection. Neutrophils isolated from Morrbid-deficient mice showed improved survival rate compared to wild-type. To examine how Morrbid regulates apoptosis of short-lived myeloid cells, neighboring genes were scanned to identify any critical regulators of apoptosis. The Bcl2l11 (Bim) gene is located within ~150kb downstream to Morrbid. Expression of Bim, but not other neighboring genes or key myeloid transcription factors, is repressed in the presence of Morrbid. On the other hand, Morrbid expression was induced by various pro-survival factors including IL-5, IL-13, and GM-CSF and thereby promoted survival of myeloid cells. Morrbid recruits polycomb repressive complex 2 (PRC2) which deposits H3K27me3 histone marks at the Bcl2l11 locus and blocks its expression (Figure 2A). Indeed, subjects with the eosinophil disorder Hypereosinophilic syndrome (HES) show significantly higher Morrbid levels than healthy counterparts, suggesting a diagnostic and therapeutic potential of this lncRNAs in inflammatory diseases. These results show the potent role of a single lncRNA in differentiation/survival of specific subsets of immune cells and the manifestation of its dysregulation in immunological disorders. 

### 2.1. A. Macrophages

Monocyte-macrophages are crucial for the modulation of physiological and pathological processes such as inflammation, tissue damage and repair, metabolism, and pathogen response. Monocytes derived from bone marrow migrate to tissues and organs along with blood circulation and then differentiate to macrophages [18]. These are dynamic and heterogeneous cells whose phenotypes and functions depend on the inflammatory signals. Currently, a variety of lncRNAs have been demonstrated to impair the function and development of monocyte-macrophages. However, knowledge of the lncRNA regulation of monocytic cell differentiation and diseases remains limited (Figure 1). In this section, we will highlight lncRNA-mediated molecular mechanisms that control macrophage differentiation, polarization, or function. 

Noncoding transcript in T cells (NTT) is a 17 kb lncRNA which is expressed in the nucleus of human primary monocytes, monocyte-derived macrophages, and the monocytic cell line THP-1 [19,20]. NTT regulates the process of inflammation in monocytes and participates in the differentiation of monocytes to macrophages. Recent studies have revealed that NTT is regulated by the monocyte key transcription factor C/EBPβ and that it binds to the promoter of nearby gene PBOV1 via hnRNP-U (Figure 2C). Overexpression of PBOV1 in THP-1 cells resulted in cell cycle arrest at the G1 stage, differentiation into macrophages, marked increase in IL-10 and CXCL10 mRNA levels, and upregulation of costimulatory molecules [21]. These findings demonstrate a multifunctional impact of NTT on macrophage differentiation and inflammatory responses suggesting their integral role in lineage commitment and acquisition of function. 

PACER, also known as COX-2-lncRNA, has been identified as a new potential target for COX-2-modulation in inflammation and cancer. This nuclear localized lncRNA is located in the upstream region of COX-2. In the PMA-induced human monocyte-macrophage differentiation system, COX-2 expression is accelerated by PACER with subsequent LPS stimulation. PACER sequesters the repressive NF-kB p50 homodimer and prevents it from binding to the promoter of COX-2, thereby facilitating formation of the active p50–p65 form of NF-kB in the promoter region of COX-2. Subsequent formation of transcription preinitiation complexes induces COX-2 transcription (Figure 2B) [22]. By regulating the COX-2 transcripts, PACER directly controls the inflammatory responses of macrophages (see Table 1).

The differentiation of monocyte to macrophage is an important branch of hematopoiesis where a broad number of important regulators are involved. A long non-coding monocytic RNA (lnc-MC) exhibit increased expression during the differentiation of THP-1 and HL-60 cells as well as CD34+ hematopoietic stem/progenitor cells (HSPCs). Lnc-MC is transcriptionally activated by PU.1, which represses miR-199a-5p expression and activates lnc-MC expression. Interestingly, PU.1, master transcriptional regulator of myeloid cell differentiation, prevents post-transcriptional silencing of lnc-MC by repressing miR-199a-5p. Transcription factors and two predominant post-transcriptional modulators, lncRNAs and miRNAs, work in concert to decisively guide myeloid cell differentiation. The upregulation of lnc-MC expression mitigates the repression of the expression of activin A receptor type 1B (ACVR1B). In immune cells, activin A signaling is critical for cell differentiation, cytokine (pro- or anti-inflammatory) production and cell migration [23,24,25]. Lnc-MC-mediated upregulation of ACVR1B expression promotes the differentiation of monocyte to macrophage [26]. These findings indicate that two different non-coding transcripts, namely lnc-MC and miR-199a-5p, act antagonistic to each other; however, a critical TF (PU-1) favors expression of lnc-MC and represses miR-199a-5p. 

Macrophages are able to differentiate and polarize into the classically activated M1 phenotype or alternatively activated M2 phenotype. Generally, M1 macrophages perform important roles in host defense and inflammation, while M2 macrophages play a part in tissue repair [27]. M1 and M2 macrophages exhibit distinct gene profiles that are regulated by specific signaling cascades, TFs, and epigenetic factors [28]. Many lncRNAs are involved in human macrophages with polarized phenotypes. A recent study in human monocyte-derived macrophage demonstrates that lncRNA TCONS_00019715 may play a critical role in promoting macrophage polarization towards a pro-inflammatory (M1) phenotype. Compared with unchallenged macrophages, expression profiling identified 9343 and 4592 differentially expressed lncRNAs in M1 and M2 macrophages. The expression of lncRNA TCONS_00019715 in IFN-γ + LPS challenged M1 macrophages is much higher than in IL-4 stimulated M2 macrophages. Additionally, the level of expression of TCONS_00019715 is increased when M2 macrophage is converted to M1 phenotype. Conversely, TCONS_00019715 expression is decreased when M1 macrophage are polarized to M2 phenotype. These data show that lncRNAs play important roles in regulating macrophage polarization [29]. Macrophage polarization is a dynamic process where cells switch phenotypes under the influence of pro- and anti-inflammatory cytokines/factors. Rapid changes in the lncRNA TCONS_00019715 can facilitate phenotype switching in macrophages. These findings clearly support a critical regulatory role of lncRNA in shaping macrophage polarization, and thus, can be utilized as novel therapeutic targets in immunomodulatory diseases. 

Obaid et al. examined expression of HOTAIR upon lipopolysaccharide (LPS) stimulation in a mouse macrophage cell line (RAW264.7) and bone marrow derived macrophages (BMDM) and observed marked induction in the levels of HOTAIR [30]. Similar to proinflammatory genes, namely, IL-6 and inducible nitric oxide synthase (iNOS), upregulation of HOTAIR is mediated by NFκB. Knockdown of HOTAIR led to the downregulation of both IL-6 and iNOS suggesting that it participates in feed-forward regulation of NFκB-mediated inflammation. Nuclear factor of kappa light polypeptide gene enhancer in B-cells inhibitor, alpha (IκBα) binds to and masks nuclear localization sequence (NLS) domain of NFκB and hence regulates its phosphorylation, nuclear translocation and activity. In LPS-stimulated macrophages, IκBα levels decrease with time, while phosphorylated NFκB increases. Knockdown of HOTAIR in LPS-treated cells mitigates NFκB activation as reflected by phosphorylation status with concomitant induction of IκBα. Treatment with MG132, a known proteasomal inhibitor, in LPS-stimulated cells prevented degradation of IκBα leading to the repression of inflammatory signaling. Compared to scramble, HOTAIR siRNA transfected cells treated with LPS and MG132 showed higher expression of IκBα suggesting that HOTAIR promotes proteasomal degradation of IκBα. 

### 2.2. Dendritic Cells

Dendritic cells (DCs) are the most potent antigen-presenting cells in mammalian immune system and the role of lncRNAs during their differentiation has an important significance during the innate and adaptive immune response. Some transcription factors, anti-inflammatory cytokines, and miRNAs have been reported to play roles in the modulation of DC function; however, the role of lncRNAs in DC function is not well understood. To date, only a handful of lncRNAs (including lnc-DC, HOTAIRM1, and MALAT1) have been shown to play a regulatory role in human DC differentiation (Figure 2D,E).

Lnc-DC is exclusively expressed in human dendritic cells and the levels of this lncRNA have been shown to increase during monocyte to dendritic cell differentiation. Lnc-DC knockdown during the differentiation of monocytes to DCs (Mo-DC) affects their capacity to uptake antigen and activate CD4^+^ T cells. In a recent study, Wang et al. showed that Lnc-DC regulates Mo-DC differentiation by directly interacting with STAT3 to prevent Y705 dephosphorylation of STAT3 by SHP1 [31] (Figure 2D). STAT3 plays a central role in DC differentiation. By maintaining activated STAT3 levels, lnc-DC controls post-translational activity of TFs involved in DC differentiation and directly contributes to the process. These results highlight that lncRNAs participate in post-translational modification that is crucial for protein activity. This study provides yet another example of the versatile functions of lncRNAs, through which they can modulate transcriptional, post-transcriptional, translational, and post-translational changes within cellular compartments.

HOTAIRM1 (HOX antisense intergenic RNA myeloid 1) is expressed in human myeloid lineage cells, located between the HOXA1 and HOXA2 genes and plays a critical role in myeloid transcriptional regulation. Using epigenetic analysis, Xin et al. examined the role of HOTAIRM1 in DC differentiation. H3K4me3 and H3K27me3 methylation ChIP-chip map analyses showed dynamic changes in the methylation pattern in the HOTAIRM1 region. LPS-induced maturation of DCs caused suppression of the transcription activation marker H3K4me3, while the inhibition marker H3K27me3 was induced. Another interesting observation was that lncRNAs interact with miRNA and mRNA to govern cellular differentiation. Authors show a potential interaction among HOTAIRM1, miR-3960, and *HOXA* genes based on the “competing endogenous (ce) RNA theory” during Mo-DC differentiation. miRNA-3960 targets both HOTAIRM1 and HOXA1 and high expression of miR-3960 could downregulate both genes and finally, promotes monocytes to differentiate into DCs [32] (Figure 2B). 

In a murine model of experimental autoimmune myocarditis (EAM), differential expression of numerous lncRNAs was observed in tolerized (sCD40L treated) and rejected cardiac allografts [33]. Among the differentially expressed lncRNAs, MALAT1 (also called NEAT2) was highly upregulated in tolerized cardiac allografts. CD11c^+^ cells were identified as the cellular reservoirs of MALAT1. The expression of MALAT1 is LPS responsive and its expression is regulated by NFκB. In DCs treated with LPS, overexpression of MALAT1 downregulated costimulatory molecules CD80, CD86, and MHCII, while MALAT1 knockdown cells exhibit antagonistic expression pattern for the same surface markers, strongly indicating that MALAT1 dampens NFκB-mediated proinflammatory signaling. Moreover, MALAT1 overexpressing DCs secrete higher anti-inflammatory (IL-10) and lower pro-inflammatory (IL-6 and IL-12) cytokines. MALAT1 enhances IL-10 production by increasing dendritic cell-specific intercellular adhesion molecule-3 grabbing nonintegrin (DC-SIGN) expression, a known regulator of IL-10. In a mixed leukocyte reaction (MLR) assay, MALAT1 overexpressing DCs reduced T-cell proliferation and enhanced T regulatory (Treg) CD4+CD25^+^ cell generation. Biochemical analysis revealed MALAT1 enrichment in Ago2 immunoprecipitation suggesting miRNA-MALAT1 interaction. Three functional miR-155 binding sites on MALAT1 were predicted and functionally validated. Interestingly, PU-1 is also a known target of miR-155. By sequestering miR-155, MALAT1 can enhance PU.1 expression, which in turn upregulates DC-SIGN (Figure 2E). Adoptive transfer of MALAT1 overexpressing DCs suppresses immune response by enhancing the Treg population and protecting mice from transplant rejection. These findings strongly support therapeutic targeting of lncRNAs in generating a tolerogenic immune environment. 

NEAT1 is another lncRNA that has been demonstrated to induce a tolerogenic phenotype in DCs [34,35]. NEAT1 is a nuclear localized lncRNA and its expression is associated with the immune response regulation in immune–mediated diseases, neurodegenerative diseases, and cancers [36,37,38,39]. NEAT1 is upregulated in response to LPS treatment and inhibits expression of costimulatory molecules CD80, CD86, and MHCII and promotes Treg generation. In this regard, NEAT1 mimics the MALAT1-medaited phenotypic impact on DC tolerance strongly supporting the notion that multiple lncRNAs work in concert to acquire a specific functional state in immune cells [33]. NEAT1 and NLRP3 are directly regulated by miR-3076-3p. NLRP3 is involved in response to injury, toxins, or invasion by microorganisms and associates with other proteins to form inflammasomes [40]. NEAT1 acts as a sponge for miR-3076-3p in DCs thereby regulating expression of NLRP3. Knockdown of NEAT1 induces a tolerogenic phenotype by suppressing NLRP3 and IL-1β levels. In the EAM model, silencing of NEAT1 inhibits disease progression by enhancing Treg generation. Adoptive transfer of NEAT1 knockdown DCs in a heart transplantation model further supported the protective role of NEAT1 in favoring immune tolerance as observed by increased Tregs in cardiac allografts, reduced Th17, and increased survival of transplant animals [34,35]. 

## 3. Lymphoid Cells 

Lymphocyte development and function is regulated by lncRNAs. As observed with other specialized immune cells, a unique repertoire of lncRNAs is expressed in lymphocytes that drives the lineage commitment by selective recruitment of transcription factors to various genes required for cell fate determination. In this section, we will provide evidence that unequivocally demonstrates the lncRNA dependence of lymphocyte differentiation as well as polarization. A list of lncRNAs and their molecular mechanism of function in lymphoid cell is listed in Table 1.

### 3.1. T Cells

#### 3.1.1. T helper (Th) Cells 

**Figure 3:**
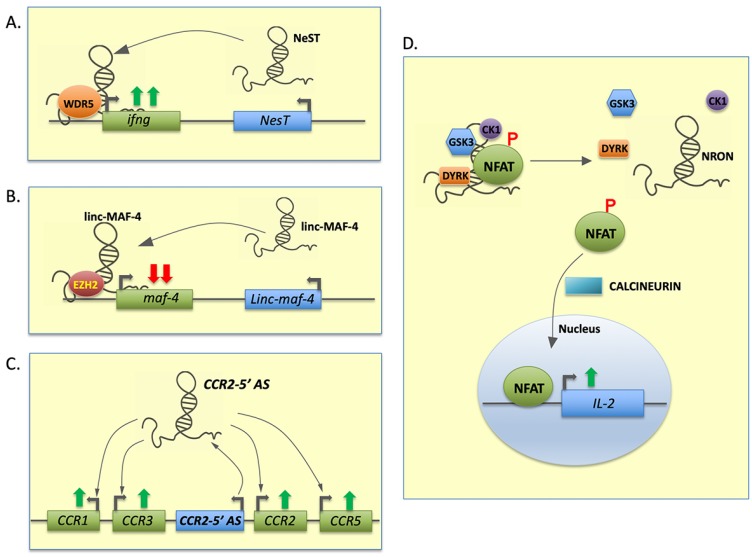
Long noncoding RNA regulates lymphoid cell differentiation and function. Multiple lncRNAs are involved in lymphoid cell differentiation and regulate polarization and effector cell functions. (**A**,**B**) NeST and linc-MAF-4 interact with specific transcription factors and guide them to the IFNG and MAF-4 locus, respectively. This directly influences transcription of target genes and promotes the Th1 phenotype. (**C**) CCR2-5’ AS induces expression of various Th2 cytokines and facilitates GATA3 (critical Th2 transcription factor) function. (**D**) In resting CD8 cells, NRON sequesters a critical transcription factor NFAT. In activated T cells, calcineurin mediates dephosphorylation and nuclear translocation of NFAT. Green arrows denote transcriptional activation and red arrows denote transcriptional repression.

Whole genome RNA sequencing (RNA-seq) analysis of human T cells differentiated under Th1, Th2 or Th17 polarizing conditions identified lncRNAs which are specifically and differentially expressed in different Th subtypes. These cell-specific lncRNAs are predominantly intergenic and located adjacent to lineage-specific protein coding genes. Th lineage-associated genes (mRNAs and lncRNAs) are not randomly located in the genome but are present in clusters. A well-characterized example of a lineage-specific lncRNA is NeST (nettoie Salmonella pas Theiler’s), which is an intergenic lncRNA located in the cluster of genes encoding IL-22 and IFN-γ in the genomic location associated with Theiler’s virus infection [41,42]. It is expressed in Th1 cells and is regulated by Th1 lineage-specific transcription factors T-bet and STAT4 [43,44]. Studies have shown that NeST expression increased only in Th1 polarized cells and not in Th2 cells and its expression correlates with IFN-γ expression. NeST regulates IFN-γ production by interacting with the methyltransferase subunit WDR5 and recruiting the transcription activation complex to the promotor of IFN-γ, resulting in the increased H3K4 methylation (Figure 3A) [45,46]. 

LncRNA TH2-LCR is another such cluster of four alternatively spliced transcripts, selectively expressed by effector Th2 lineage. TH2-LCR is located in the IL-4, IL-5, and IL-13 gene cluster. It is co-expressed with them and regulates the expression of these Th2 cytokines in a manner similar to NeST. Knockdown of TH2-LCR resulted in decreased binding of WDR5 on IL-13 and IL-4 promoters as well as reduced H3K4 methylation [47,48]. LincR-Ccr2-5′AS has also been identified as a cell-specific lncRNA in Th2 cells. Expression of this lncRNA is regulated by GATA3 transcription factor, an important regulator of Th cell development. Knockdown of lincR-Ccr2-5′AS dysregulated the expression of nearly 1200 genes, a majority of which are also regulated by GATA3 (Figure 3C) [49]. These findings strongly suggest that lncRNAs work in concert with critical transcription factors and facilitate their activity by regulating similar set of downstream genes targets to acquire cellular phenotype and function.

LncRNA-MAF-4 has been shown to play important role in Th1/Th2 polarization [50,51]. It is highly expressed in Th1 cells, but the expression is significantly lower in Th2 cells. LncRNA-MAF-4 interacts with the MAF promoter and EZH2 (Enhancer of Zeste 2 Polycomb Repressive Complex 2 Subunit) and facilitates the binding of the chromatin-modifying complex and increases H3K27 tri-methylation at the MAF promoter, leading to transcriptional repression [50]. Knockdown of lncRNA-MAF-4 increases the expression of MAF4 and other Th2-specific genes in naïve CD4^+^ T cells [50]. Another lncRNA, lncRNA-CD244, regulates the expression of IFN-γ and TNF-α by a similar mechanism in CD8^+^ T cells [52]. LncRNA-CD244, upon induction through CD244 (T cell inhibitory receptor), binds to EZH2 and recruits it to the promoters of IFN-γ and TNF-α and represses their transcription (Figure 3B). Knockdown of lncRNA-CD244 in CD8^+^ T cells enhances their function [52].

#### 3.1.2. CD8+ T Cells

NFAT1 (Nuclear factor of activated T cells), a calcium-dependent transcription factor, plays a major role in the proliferation and differentiation of naïve T cells into specific effector subsets. NFAT together with AP-1 and NFκB regulate the production of IL-2. Production of IL-2 and the switch from low- to high-affinity IL-2 receptor promotes the differentiation and proliferation of naïve T cells. Using sh-RNA based in vitro screening, Willingham et al. identified an intronic lncRNA NRON (noncoding repressor of NFAT) as a repressor of NFAT1 [53]. NRON sequesters NFAT1 in the cytoplasm and blocks its translocation to the nucleus. In resting T cells, NRON together with another scaffold protein IQGAP (IQ motif containing GTPase activating protein) and three inhibitory kinases DYRK (dual specificity tyrosine phosphorylation regulated kinase), GSK-3 (glycogen synthase kinase 3) and CK1 (casein kinase 1), binds and sequesters the phosphorylated NFAT1 in the cytoplasm. In activated T cells, calcineurin dephosphorylates NFAT1, which is then translocated to nucleus [54,55]. Knockdown of NRON disrupts this RNA-protein complex and results in the increased dephosphorylation and nuclear translocation of NFAT1 and T cell activation (Figure 3D). Disruption of this complex by other methods also leads to similar results as reported in IQGAP KO mice. CD8^+^ cells from these mice produce significantly higher levels of IFN-γ, which is a well-known downstream target of NFAT1. Loss of LRRK2 (Leucine Rich Repeat Kinase 2), a kinase which stabilizes the NFAT1-NRON complex also leads to increased NFAT1 nuclear translocation and induction of downstream genes. Mice deficient in LRRK2 manifest aggravated clinical symptoms as well as increased proinflammatory cytokine production in experimentally induced colitis [56].

#### 3.1.3. Regulatory T cells (Tregs)

Regulatory T cells (Tregs) are a subtype of T cells that regulate or suppress other immune cells and control the immune response, thus helping to maintain homeostasis and self-tolerance and prevent autoimmune diseases. Foxp3 is the most specific marker of Tregs [57]. LncRNA FLICR (Foxp3 long intergenic noncoding RNA) negatively regulates the expression and function of Foxp3 in Foxp3^+^ T cells [58]. FLICR is only expressed in mature Tregs and decreases the expression of Foxp3 to 2–5-fold. FLICR acts only in cis, and it does not affect DNA methylation but modifies chromatin accessibility by targeting a repressive complex to the CNS3/AR5 region of Foxp3 [56]. CNS3 (Conserved Non-coding Sequence 3) is an important enhancer element required for stable Foxp3 expression. Brajic et al. identified the novel Treg-specific lncRNA Foxp3-specific lncRNA anticipatory of Tregs (FLATR) as a potential upstream regulator of Treg conversion [59]. However, FLATR KO mice did not exhibit marked impact on Treg induction.

### 3.2. B Cells

B cell differentiation starts in the bone marrow from Hematopoietic Stem Cells (HSCs), which differentiate to multipotent progenitor (MPP) cells and subsequently to common lymphoid progenitor (CLP) cells. CLPs undergo several differentiation stages namely pro-B cells, pre-B cells, and immature B cell stages. Each stage is marked by specific gene expression signatures and immunoglobulin Heavy (H) and light (L) gene loci arrangement [60,61,62]. Several studies have reported stage-specific expression of lncRNAs during B-cell development. Using 11 distinct B-cell subsets including pre-B1 cells, pre-B2 cells, immature B cells, naïve B cells, and memory B cells, Petri et al. identified several lncRNAs which were associated with differentiation-specific gene networks. Among these, lncRNAs LEF-AS1, MYB-AS1, and SMAS-AS1, expressed predominantly in pre-B1 and pre-B2, originate from protein-coding genes with known function in B cell development and are also reported to be expressed in various stages of B-cell development [63].

PAX5 (paired box 5) is an important transcription factor in B-cell development [64]. Studies in mice have revealed that PAX5 regulates the expression of several lncRNAs which also interact with PAX5. PAIR4 (PAX5-activated intergenic repeat 4) and PAIR6 (PAX5-activated intergenic repeat 6) are transcribed as antisense to PAX5 and are expressed in progenitor B-cells during V(D)J recombination. They play a pivotal role in locus compaction and V(D)J recombination, a critical phenomenon that confers diversity to the adaptive arm of immune cells [65].

CRNDE (colorectal neoplasia differentially expressed) is a bidirectional lncRNA expressed in the proliferative stages of B cell development. CRNDE and several other lncRNAs were found to be co-expressed with mitotic cell cycle genes in centroblasts in germinal cells as well as precursor B-cells [63].

### 3.3. Natural Killer Cells

Natural killer (NK) cells are a subtype of type 1 innate lymphoid cells that functions as a first line of innate immune defense. NK cells are characterized as CD56 positive and CD3 negative, secrete perforin, TNF-α, and IFN-γ and perform cell-mediated cytotoxicity [66]. CD56 is an important marker of NK cells and based on CD56 expression levels NK cells are characterized as CD56 bright (mainly produce pro-inflammatory cytokines) and CD56 dim (mainly cytotoxic) [67,68,69,70]. Lnc-CD56 is an intronic lncRNA, transcribed from the first intron of the CD56 gene that positively regulates the expression of CD56. Zhang et al. showed that shRNA-mediated knockdown of lnc-CD56 results in the decreased expression of CD56, which suggests its role in NK cell differentiation [71]. LncRNA IFNG-AS1 also known as NeST or TMEVPG1 is upregulated in NK cells upon stimulation and increase the production of IFN-γ [72]. However, the detailed molecular mechanisms underlying the role of lncRNAs in NK cell functions demands further investigation.

## 4. LncRNAs in Immune-Related Diseases

Considering their diverse molecular functions in the regulation of wide spectrum of cellular processes, it is not surprising that lncRNAs have been associated with disease. Several studies have reported that lncRNAs are dysregulated in disease [73]. Genome-wide association studies (GWAS) have linked various single nucleotide polymorphisms (SNPs) to immune-related diseases [74]. Approximately 10% of these disease-associated SNPs were mapped to the genomic locations encoding lncRNAs [75,76]. In addition, several recent studies have also demonstrated the role of lncRNAs in immune-related diseases. LncRNAs HOTAIR and H19 were found to be upregulated in rheumatoid arthritis [77,78]. Shi et al. have reported that expression of several lncRNAs were significantly altered in PBMCs isolated from Systemic lupus erythematosus (SLE) patients. Two lncRNAs located on chromosome 6 and surrounded by significantly deregulated genes such as T cell activation RhoGTPase activating protein (TAGAP), Wilms tumor 1 associated protein (WTAP), superoxide dismutase 2 (SOD2), acetyl-CoA acetyltransferase 2 (ACAT2), and fibronectin type III domain containing 1 (FNDC1) were highly upregulated. A lncRNA located upstream of Human Immunodeficiency Virus Type I Enhancer Binding Protein 2 (HIVEP2) was also significantly upregulated [79]. Another study by Hrdlickova et al. found lncRNA enrichments in several immune-related diseases such as celiac disease, primary biliary cirrhosis, psoriasis, primary sclerosing cholangitis, inflammatory bowel disease, juvenile idiopathic arthritis, and rheumatoid arthritis [80]. Overall, these findings strongly suggest that lncRNAs may be important drivers in immunomodulatory diseases. Identifying the cellular reservoirs contributing to transcriptional changes in lncRNA expression will further advance our understanding of their role in the disease pathogenesis. With the significant progress in the field of CRISPR-Cas-based genomic modification, approaches to target cell-specific changes in lncRNA may facilitate development of novel treatment modalities for immune-cell mediated diseases.

## 5. Concluding Remarks

The advances in high throughput sequencing enabled us to carry out transcriptome wide identification of the cellular and tissue expressed lncRNA repertoire, identifying thousands of novel and differentially expressed lncRNAs in various immune cell types and diseases. The next big challenge is to assign a function to these lncRNAs and dissect the molecular mechanism of their action in the context of cellular homeostasis and disease pathogenesis. Immune cells, particularly in inflamed tissues, are under an unstable environment and have to adapt and efficiently perform both pro-and anti-inflammatory functions. It will be interesting to convincingly demonstrate in vivo that the expression profiles of polarization markers correlate with the lncRNAs.

In recent years lncRNAs have emerged as new but potent modulators of gene expression in diverse lineages of cell and tissue types, regulating almost all aspects of cellular function. A consistent observation is that lncRNA functions are also cell specific. Their ability to attain and adapt structures compatible to various proteins/protein complexes present in different cell types and/or stimuli allows lncRNA to rapidly switch cellular phenotype. Similarly, long sequences and various lncRNA isoforms increases the likelihood of binding multiple miRNAs and compete with them for their functional targets, thereby contributing to the changes in translational output. These molecular features of lncRNA are particularly pertinent to immune cells that exhibit dynamic functional plasticity in a local microenvironment. A comparison of global lncRNA, protein-coding RNA, and microRNA profiles during immune cell differentiation will provide holistic and comprehensive information on lncRNA-mediated regulation of this dynamic process and their interaction with other protein-coding and noncoding RNAs.

LncRNAs are distributed throughout the cell including the cytoplasm, mitochondria, ribosomes, nucleus, and chromatin speckles. They perform diverse molecular functions in these subcellular locations. Similar to proteins, the specific molecular functions of lncRNA may depend and change based on their subcellular localization. The localization of lncRNAs is not always exclusive to a particular organelle or subcellular domain. For example, lncRNA RMRP is present in the nucleus, cytoplasm, and mitochondria [81,82]. Additionally, the subcellular localization of lncRNAs can be dynamic and controlled by a specific signal. LncRNA SNHG1 is present in both the nuclear and cytoplasmic compartments in HCT116 cells (colon cancer cells) in normal conditions, but are exclusively retained in the nucleus upon doxorubicin-mediated DNA damage [83]. LncRNA Uchl1-AS1 shuttles from the nucleus to the cytoplasm upon treatment with rapamycin and promotes translation of Uchl1 mRNA [84]. It would be interesting to study lncRNA localization in the context of immune cell differentiation and function.

## Figures and Tables

**Table 1:** Long noncoding RNAs involved in the development and function of myeloid and lymphoid cells.

Myeloid Cells			
LncRNA	Cell Type	Function	Refs.
**Morrbid**	Myeloid cells	Controls the survival of short-lived myeloid cells by *cis* regulation of Bcl2l11 expression	Kotzin, 2016
**NTT**	Monocytes	Binds to promoter of PBOV1 via hnRNP-, U promotes cell cycle arrest, differentiation into M0M2, increase in IL-10, CXCL10 mRNA levels, and upregulation of the costimulatory molecules	Yang, 2018
**PACER**	Monocytes	Binds to and titrates the repressive NF-B1 homodimer away from the COX2 promoter, thereby facilitating binding of the activating RELA/NF-B1 heterodimer and subsequent formation of transcription preinitiation complexes	Krawczyk, 2014
**Lnc-MC**	Monocytes	Facilitates the differentiation of monocytes by enhancing the effect of PU.1 and sequestering miR-199a-5p and increasing the expression of ACVR1B	Chen, 2015
**TCONS_00019715**	Monocytes	Promotes macrophage polarization towards pro-inflammatory (M1) phenotype	Huang, 2016
**HOTAIR**	Monocytes	Enhances proinflammatory NFκB signaling by promoting IκBα degradation	Obaid, 2018
**Lnc-DC**	Dendritic cells	Promotes STAT3 signaling by interacting with the C terminus of STAT3 to prevent the dephosphorylation of STAT3 Y705 by SHP1	Wang, 2014
**HOTAIRM1**	Dendritic cells	Promotes monocyte/dendritic cell differentiation through competitively binding to endogenous miR-3960	Xin, 2017
**NEAT1**	Dendritic cells	Induces tolerogenic phenotype in DC and promotes Treg polarization by inhibiting NLRP3 via sequestering miR-3076-3p	Zhang, 2019 Wu, 2017
**Malat1**	Dendritic cells	Induces tolerogenic phenotype in DC and promote Treg polarization by sponging miR-155 and upregulating PU.1 expression	Wu, 2018
**Lymphoid Cells**			
**LncRNA**	**Cell Type**	**Function**	**Refs.**
**NeST**	CD8+ T cell and TH1 cell	Binds to WDR5 and regulate the expression of IFN-gamma by recruiting transcription activation complex to IFN-gamma promoter	Gomez, 2013 Collier, 2014
**NRON**	T cell	Sequester phosphorylated NFAT in cytoplasm of resting T cells	Willingham, 2005; Okamura, 2002; Sharma, 2001
**TH2-LCR**	TH2 cell	Regulates the expression of TH2 cell cytokines, including IL-4, IL-5, and IL-13	Koh, 2010; Spurlock, 2015
**LncRNA-CD244**	CD8+ T cell	Inhibits expression of IFNG and TNF by recruiting EZH2 to their promoters	Wang, 2015
**Linc-MAF-4**	TH1 cell	Regulates the expression of MAF and promote TH1 differentiation	Ranzani, 2015 Zhang, 2017
**LincR-Ccr2-5′AS**	TH2 cell	Regulate the expression of TH2 cytokines	Hu, 2013
